# Short-term mycophenolic acid withdrawal is associated with temporal changes in immune cell composition in mRNA-1273-vaccinated kidney transplant recipients: insights from high-dimensional immune profiling

**DOI:** 10.3389/fimmu.2026.1826672

**Published:** 2026-08-17

**Authors:** Nina Goerlich, Maximilian Stich, Manina Günter, Tessa Kühn, Claudius Speer, Matthias Schaier, Burkhard Tönshoff, Martin Zeier, Christian Morath, Miguel Fribourg, Stella E Autenrieth, Louise Benning

**Affiliations:** 1 Department of Nephrology and Medical Intensive Care, Charité-Universitätsmedizin, Berlin, Germany; 2 Berlin Institute of Health, Charité – Universitätsmedizin, Berlin, Germany; 3 Department of Pediatrics I, Center for Pediatric and Adolescent Medicine, Medical Faculty Heidelberg, Heidelberg University, Heidelberg, Germany; 4 German Cancer Research Center (DKFZ), Division Virus-Associated Carcinogenesis, Heidelberg, Germany; 5 German Center for Infection Research (DZIF), Heidelberg Partner Site, Heidelberg, Germany; 6 Dendritic Cells in Infection and Cancer (D431), German Cancer Research Center (DKFZ), Heidelberg, Germany; 7 Department of Nephrology, Heidelberg University Hospital, Heidelberg University, Heidelberg, Germany; 8 Department of Nephrology and Hypertension, Klinikum Nuremberg, Paracelsus Medical University, Nuremberg, Germany; 9 Institute for Translational Medicine and Pharmacology, Department of Pharmacological Sciences, Icahn School of Medicine, New York, NY, United States; 10 Immunology Institute, Icahn School of Medicine, New York, NY, United States

**Keywords:** high-dimensional flow cytometry, immune cell composition, immunosuppression, kidney transplantation, mycophenolate mofetil

## Abstract

**Background:**

Short-term mycophenolic acid (MPA) withdrawal is common in kidney transplant recipients with severe infections and was extended during the COVID-19 pandemic to improve vaccine immunogenicity, yet its effects on immune cell dynamics remain mechanistically understudied.

**Methods:**

We performed high-dimensional flow cytometry combined with unsupervised computational clustering to analyze longitudinal peripheral blood mononuclear cell samples from kidney transplant recipients who received mRNA-1273 vaccination. Samples were collected before and after a 5-week temporary MPA discontinuation, and the results were compared with those of vaccinated patients maintained on standard triple immunosuppression.

**Results:**

Compared with continued triple immunosuppression (N = 13), MPA withdrawal (N = 11) was associated with temporal shifts in major immune compartments, including increases in the frequencies of CD4^+^ and CD8^+^ T cells (*P* = 0.039 and *P* = 0.001, respectively), as well as decreases in the frequencies of monocytes (*P* = 0.014) and B cells (*P* = 0.023). These changes were accompanied by increased relative frequencies of specific T-cell subsets, including CD8^+^ TEMRA CD38^+^ cells (*P* = 0.011) and early activated memory CD4^+^ T cells (*P* = 0.023). With increasing time since transplantation, these longitudinal patterns attenuated. Older patient age was associated with lower overall monocyte frequencies, though it did not influence their temporal trajectories. Although exploratory analyses showed subset differences between seroconverters and non-seroconverters after vaccination, no major compartment or individual subset was predictive in regression models.

**Conclusions:**

Short-term MPA withdrawal was associated with selective temporal immune shifts, including increased relative frequencies of CD4^+^/CD8^+^ T-cell subsets and decreased relative frequencies of monocyte and B-cell subsets. Effects varied by time since transplantation and age, reflecting heterogeneous immune reconstitution. High-dimensional profiling enabled detailed analysis of major and rare immune populations, highlighting its value in mechanistic transplantation studies.

## 1 Introduction

Lifelong immunosuppression is essential after kidney transplantation to prevent rejection, the leading cause of late allograft loss (1, 2). The remarkable progress in transplantation and the significant reduction in rejection rates can mainly be attributed to major advances in immunosuppressive therapies (3). Recent OPTN/SRTR data report that a triple combination therapy of tacrolimus, mycophenolic acid (MPA), and steroids is the most commonly used regimen following adult kidney transplantation (68.8%), followed by a dual therapy of tacrolimus and MPA without steroids (24.7%) (4). Combination therapy is favored because it improves efficacy through the cumulative effects of targeting multiple T−cell pathways while allowing dose minimization to reduce drug-related toxicity (3, 5).

The pharmacological basis of current immunosuppressive regimens lies in the central role of T-cell activation in graft rejection, which is classically described as requiring three coordinated signals: antigen recognition (signal 1), costimulatory engagement (signal 2), and cytokine−driven proliferation (signal 3) (6). Calcineurin inhibitors (CNIs) block the calcium–calcineurin pathway, which is activated downstream of T−cell receptor and costimulatory signaling (signals 1 and 2) (6). By preventing activation of the transcription factor nuclear factor of activated T cells (NFAT), CNIs suppress interleukin−2 production and limit the clonal expansion of T cells (6). In parallel, MPA inhibits the inosine monophosphate dehydrogenase, the rate−limiting enzyme in *de novo* guanosine nucleotide synthesis, thereby impairing the *de novo* nucleotide synthesis essential for signal 3–mediated lymphocyte proliferation and differentiation (6). Glucocorticoids exhibit broad immunosuppressive, anti-inflammatory, and lympholytic effects by reducing cytokine production and lymphocyte proliferation (7). Taken together, these different agents synergistically suppress both the activation and expansion of alloantigen−reactive lymphocytes.

The downside of potent immunosuppression is the increased susceptibility to infections, a leading cause of death among transplant recipients (8). To minimize this risk, structured immunosuppression minimization strategies have been, and continue to be, investigated in various clinical trials (9–14), often tailored to individual immunological risk (15). Independent of such protocols, short−term MPA withdrawal is commonly practiced in hospitalized patients with infections, as its antiproliferative effects can impair viral and bacterial control (16). During the COVID−19 pandemic, this strategy was extended to vaccination, with temporary MPA withdrawal or reduction in vaccination non−responders showing promising improvements in seroconversion rates compared with those who continued triple immunosuppressive therapy (17–21).

Building on this experience, short−term MPA withdrawal was also implemented at our center to enhance vaccine immunogenicity (20, 21), providing a unique opportunity to systematically investigate immune cell dynamics in response to MPA withdrawal within a carefully designed study. In this sub-study, we applied high-dimensional immune phenotyping to assess how short-term MPA withdrawal affects the immune landscape in kidney transplant recipients (KTR), aiming to understand the cellular changes associated with this clinically relevant, yet mechanistically poorly understood, intervention.

## 2 Materials and methods

### 2.1 Study population

This study was conducted as part of a well-characterized prospective cohort designed to assess vaccine immunogenicity in KTR who remained seronegative after ≥3 SARS-CoV-2 vaccinations (non-responders) (20, 21). In the main study, vaccine response was assessed during short-term MPA withdrawal as a strategy to improve vaccine immunogenicity in selected KTR. MPA withdrawal was offered to patients receiving triple immunosuppressive maintenance therapy (CNI, MPA, and corticosteroids), with stable graft function (serum creatinine ≤2.5 mg/dL and proteinuria ≤200 g/mol creatinine) and no biopsy-proven graft rejection within the previous 12 months.

For this analysis, a subset of the original cohort was included. Of these, 11 KTRs underwent short-term MPA withdrawal, initiated one week before vaccination and continued for 4 weeks after vaccination. The remaining patients continued standard triple immunosuppressive therapy throughout the study period. All patients received a 100 µg dose of the mRNA-1273 vaccine (Moderna, Cambridge, Massachusetts, USA). Peripheral blood was collected at three predefined time points: T0 (pre-vaccination/pre-withdrawal), T1 (1 month post-vaccination; end of 5-week MPA withdrawal), and T2 (3 months post-vaccination; after MPA reintroduction). Five healthy controls were included as reference comparators but excluded from longitudinal analyses due to single-timepoint sampling only. Immune phenotyping was performed on peripheral blood mononuclear cells (PBMCs) isolated from all collected samples.

All study participants gave written informed consent. The study was conducted in accordance with the Declaration of Helsinki, approved by the ethics committee of Heidelberg University, and registered in the German Clinical Trials Register (DRKS00024668).

### 2.2 PBMC isolation and preparation

PBMCs were isolated from peripheral blood by Ficoll density gradient centrifugation, cryopreserved, and stored in liquid nitrogen at the Department of Nephrology at Heidelberg University Hospital (see **Supplementary Methods**). PBMC staining, measurement, unmixing, and QC analysis were performed at the German Cancer Research Center (DKFZ) in Heidelberg. Cryopreserved PBMCs were thawed rapidly in a 37 °C water bath, followed by resuspension in 10 mL RPMI medium (MilliporeSigma; Billerica, MA, USA) containing 50 KU DNase I (Merck, Darmstadt, Germany) to minimize cell clumping. After centrifugation (400 × g, 5 minutes, room temperature), the pellet was resuspended in 5 mL RPMI with 200 KU DNase I and incubated for 20 minutes at 37 °C.

Cell viability was assessed by trypan blue exclusion. After washing once with PBS, 3 million viable PBMCs were prepared per sample. Dead cells were excluded using the LIVE/DEAD Fixable Blue Dead Cell Stain Kit (Thermo Fisher Scientific). To block Fc receptor-mediated non-specific antibody binding, Human TruStain FcX (BioLegend, San Diego, CA, USA) was added prior to staining.

Extracellular staining was performed at 4 °C for 1 hour using a validated 36-color antibody panel (**Table 1**) (22), including True Stain Monocyte Blocker (BioLegend) to prevent non-specific binding of selected fluorochromes to monocytes. After two washes, samples were resuspended for cytometric analysis.

**Table 1:** Antibody panel used for high-dimensional flow cytometry of PBMCs.

Antibody	Company	Catalog number	Clone	Concentration
CD1c AlexaFluor647	BioLegend	331510	L161	1.25 µg/ml
CD3 BV510	BioLegend	317332	OKT03	0.75 µg/ml
CD4 cFluorYG584	Cytek Biosciences	R7-20041	SK3	0.25 µg/ml
CD8 BUV805	BD Bioscience	612889	SK1	0.8 µg/ml
CD11b PerCP-Cy5.5	BioLegend	301338	ICRF44	5 µg/ml
CD11c BUV661	BD Bioscience	612967	B-ly6	1.25 µl in 100 µl
CD14 Spark Blue 550	BioLegend	367148	63D3	1.6 µg/ml
CD16 BUV496	BD Bioscience	612944	3G8	0.6 µg/ml
CD19 Spark NIR 685	BioLegend	302270	HIB19	0.6 µg/ml
CD20 Pacific Orange	Thermo Fisher Scientific	MHCD2030	HI47	1.25 µl in 100 µl
CD24 PE Dazzle594	BioLegend	311134	ML5	2 µg/ml
CD25 PE	BioLegend	302606	BC96	0.75 µg/ml
CD27 APC	BioLegend	356410	M-T271	2.5 µg/ml
CD28 BV650	BioLegend	302946	CD28.2	1.5 µg/ml
CD38 APC Fire810	BioLegend	102746	HIT2	0.75 µg/ml
CD45 PerCP	BioLegend	368506	2D1	2 µg/ml
CD45RA BUV395	BD Bioscience	740315	5H9	2 µg/ml
CD56 BUV737	BD Bioscience	612766	NCAM16.2	2.5 µl in 100 µl
CD57 FITC	BioLegend	359604	HNK-1	0.6 µg/ml
CD95 PE/Cy5	BioLegend	305610	DX2	1.2 µg/ml
CD123 Super Bright436	Thermo Fisher Scientific	62-1239-42	6H6	0.3 µg/ml
CD127 APC-R700	BD Bioscience	565185	HIL-7R-M21	1.25 µl in 100 µl
CD141 BrilliantBlue515	BD Bioscience	566017	1A4	3 µl from a 1:5 pre-dilution in 100 µl
CD161 eFluor450	Thermo Fisher Scientific	48-1619-42	HP-3G10	1.25 µg/ml
CD183 (CXCR3) PE/Cy7	BioLegend	353720	G025H7	2 µg/ml
CD185 (CXCR5) BV750	BD Bioscience	747111	RF8B2	1.2 µg/ml
CD195 (CCR5) BUV563	BD Bioscience	741401	2D7/CCR5	5 µg/ml

Overview of the antibodies, manufacturers, catalog numbers, clones, and concentrations used for immune phenotyping by spectral flow cytometry. Antibodies were selected to cover major T cell, B cell, NK cell, monocyte, and dendritic cell subsets. Concentrations refer to the final antibody amount used per staining volume.

Longitudinal samples were thawed and stained simultaneously to reduce batch effects.

### 2.3 High-dimensional flow cytometry and data analysis

The samples were measured on a Cytek Aurora spectral flow cytometer (Cytek Biosciences, Frankfurt, Germany), equipped with five lasers (UV 355 nm, Violet 405 nm, Blue 488 nm, Yellow-Green 561 nm, Red 638 nm), three scattering channels, and 64 fluorescence channels spanning 365–829 nm. Spectral unmixing was performed in SpectroFlo 3.1.0 using bead-based single-stain controls, except for live/dead, CD45RA-BUV395, CD56-BUV737, CD141-BB515, TCRgd-PerCP-eFluor710, and CD24-PE-Dazzle. Unstained PBMCs were used for autofluorescence subtraction. PBMCs from the same buffy coat served as batch controls to monitor instrument stability and batch effects across acquisitions.

To analyze high-dimensional spectral flow cytometry data, we employed an unsupervised clustering approach based on the FlowSOM algorithm (23), which enables robust, unbiased identification of immune cell populations.

Initially, labeled cells from all patients were computationally pooled to identify major immune compartments. FlowSOM clustering was performed on this *in silico* pooled dataset, and the resulting 17 metaclusters were manually curated and annotated. FlowSOM clustering results, including the spanning tree, metacluster assignments, and marker-expression profiles used for manual annotation, are provided in **Supplementary Figures 1**, **2**. The reproducibility of the repeated clustering analyses is summarized by the coefficients of variation (CVs) of the annotated immune cell populations in **Supplementary Table 1**. Subsequently, the frequencies of shared cell populations were determined by summing the relevant metacluster frequencies and then by debarcoding to assign values to individual patients. To provide an exploratory assessment of longitudinal changes in immune cell numbers, leukocyte-scaled cell-count estimates were calculated by multiplying the relative frequency of each major immune cell population by the corresponding total peripheral leukocyte count obtained from routine clinical laboratory measurements (**Supplementary Figure 3**). Because flow cytometric analyses were performed on isolated PBMCs, whereas total leukocyte counts include granulocytes, these estimates do not represent validated absolute immune cell counts and were interpreted as exploratory analyses only.

To ensure consistency and reduce measurement bias, the following steps were implemented:

1. Clustering on pooled samples: by clustering the pooled dataset and then demultiplexing, we ensured consistent subset mapping across all samples, improving comparability between individuals.
2. Equal cell representation: to prevent clustering bias toward samples with higher numbers of specific immune subsets (CD4^+^ T cells, CD8^+^ T cells, monocytes, B cells), we standardized the input by randomly downsampling each sample to match the sample with the lowest cell count within the panel of interest. The total number of cells available per sample and the number of cells included in each FlowSOM analysis are provided in **Supplementary Table 2**.
3. Repetition for low-abundance subsets: to increase the reliability of detecting rare populations, the sampling and clustering steps were repeated up to three times. Results were averaged to enhance robustness for low-frequency subsets.

No significant variation in marker signal intensity or in the frequencies of major immune compartments was observed across batches, indicating minimal batch effect.

We used unsupervised clustering to analyze high-dimensional flow cytometry data due to its ability to handle high-dimensional datasets, reduce human bias, and identify rare or novel cell populations that may be missed by traditional gating strategies. This approach is widely accepted by the scientific community and has been validated in numerous studies, including those from our group, making it a robust tool for comprehensive immune profiling (24, 25).

To analyze longitudinal changes in immune cell frequencies, linear mixed-effects models were fit using the lme4 package in R (26). Patient ID was included as a random effect to account for repeated measures. Between-group comparisons (MPA withdrawal vs. continued standard immunosuppression) included fixed effects for timepoint, immunosuppression group, and their interaction. Separate within-group models with timepoint as the only fixed effect were used to assess temporal changes within each group independently. *P*-values for fixed effects were obtained from Type III ANOVA tables with Satterthwaite’s approximation (lmerTest package). Significant overall timepoint effects were further examined using *post hoc* pairwise contrasts of estimated marginal means (emmeans package) with Tukey adjustment for multiple testing. Additionally, to investigate the influence of patient-specific characteristics on immune trajectories, interaction models were used within the MPA withdrawal group, incorporating timepoint and clinical covariates such as time since transplantation, and patient age. To examine whether changes in immune cell frequencies were associated with vaccine-induced seroconversion, logistic regression models were applied. *P*-values for fixed effects in the mixed-effects models were obtained using Satterthwaite’s approximation via the lmerTest package. To identify immune cell subsets associated with vaccine-induced seroconversion at timepoint 1, we analyzed all major immune cell compartments and their corresponding immune cell subsets using two-sided Mann–Whitney U tests for unpaired comparisons. To account for multiple testing across all evaluated immune cell subsets, p values were adjusted using the Benjamini–Hochberg false discovery rate (FDR) procedure. Model assumptions were verified by visual inspection of residuals.

Computational processing and statistical analyses were conducted at Charité – Universitätsmedizin Berlin. Due to variability in sample quality and limited sample size, analyses remain mainly descriptive, with statistical evaluations included but interpreted cautiously.

## 3 Results

### 3.1 Baseline characteristics

A total of 69 KTR were enrolled between January 11, 2022, and February 18, 2022, at the Department of Nephrology, Heidelberg University Hospital. For this sub-analysis, 11 patients who underwent short-term MPA withdrawal and 13 patients who continued standard triple immunosuppression were included. Blood was collected at predefined timepoints with T0 (pre-vaccination/pre-withdrawal), T1 (1 month post-vaccination; end of 5-week MPA withdrawal), and T2 (3 months post-vaccination; after MPA reintroduction). Baseline demographics for the full cohort are summarized in **Table 2**.

**Table 2:** Baseline characteristics.

Characteristics of kidney transplant recipients on CNI+MPA+steroids N=24	MPA withdrawal N=11	MPA continuation N=13
Basic patient characteristics		
Female, N (%)	4 (36)	6 (46)
Age at t0, median (IQR)	59 (52–66)	59 (47–63)
BMI (kg/m^2^), median (IQR)	25 (22–31)	23 (21–28)
Transplant data		
Time since transplantation (years), median (IQR)	2.3 (1.6–5.7)	4.5 (1.4–5.9)
Donor characteristics		
Deceased, N (%)	7 (64)	11 (85)
Living, N (%)	4 (36)	2 (15)
S-Creatinine at t0 (mg/dl), median (IQR)	1.27 (1.19–1.43)	1.55 (1.18–1.80)
S-Creatinine at t1 (mg/dl), median (IQR)	1.27 (1.11–1.52)	1.49 (1.02–1.80)
S-Creatinine at t2 (mg/dl), median (IQR	1.30 (1.09–1.47)	1.36 (1.20–1.92)
TAC trough level at t0 (µg/l), median (IQR)	8.4 (6.0–8.6)	7.3 (5.7–7.9)
Primary kidney diease		
Glomerular disease, N (%)	7 (64)	2 (15)
PKD, N (%)	2 (18)	2 (15)
Reflux/chronic pyelonephritis, N (%)	0 (0)	1 (8)
Vascular, N (%)	0 (0)	1 (8)
Diabetes, N (%)	0 (0)	3 (23)
Other/Unknown, N (%)	2 (18)	4 (31)
Comorbidities		
Arterial hypertension, N (%)	8 (73)	8 (62)
Diabetes, N (%)	1 (9)	3 (23)
CAD, N (%)	5 (45)	5 (38)
Chronic lung disease, N (%)	4 (36)	1 (8)
Chronic liver disease, N (%)	0 (0)	2 (15)
Malignancy, N (%)	4 (36)	2 (15)

BMI, body-mass index; CAD, coronary artery disease; CNI, calcineurin inhibitor; IQR, interquartile range; IS, immunosuppression; MPA, mycophenolic acid; N, number; PKD, polycystic kidney disease; TAC, tacrolimus.

### 3.2 Frequencies of CD4^+^ and CD8^+^ T cells increase, while frequencies of monocytes and B cells decrease in patients undergoing MPA withdrawal

**Figure 1:**
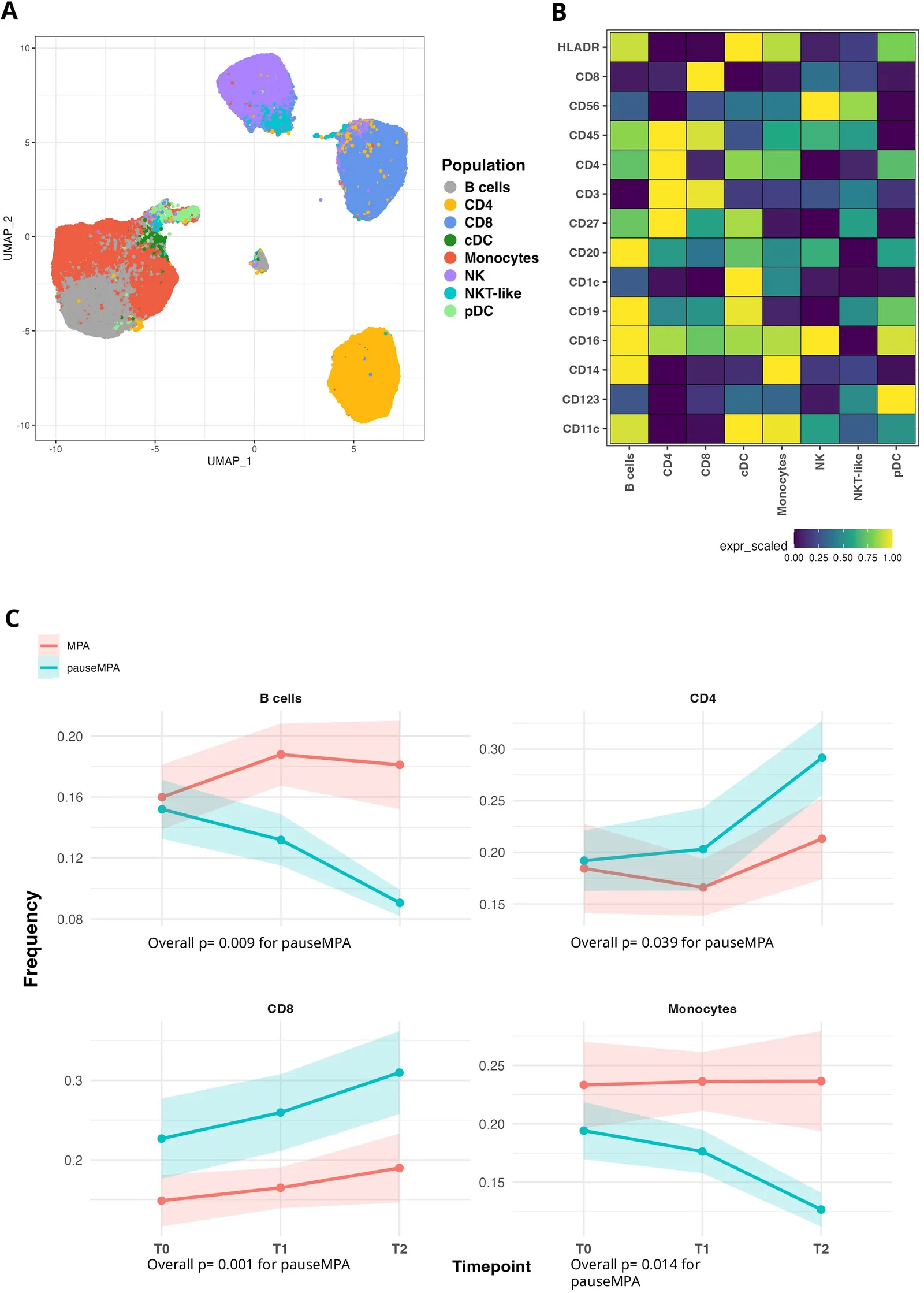
Major immune cell compartments in patients undergoing MPA withdrawal or continued triple immunosuppressive therapy. **(A)** Uniform manifold approximation and projection (UMAP) visualization of high-dimensional flow cytometry data. The obtained 17 metaclusters were assigned to 8 major immune cell compartments. **(B)** Heatmap of marker expression profiles used to define the 8 major immune cell compartments **(C)** Longitudinal trajectories (mean and SEM for each group at each timepoint) of B cells, T cells, and monocytes, with MPA-continuation patients in red and MPA-withdrawal patients in blue. Only p values from the within-group longitudinal mixed-effects models (assessing changes in trajectories over time within each immunosuppression group) are shown. Between-group comparisons are described separately in the Results. cDC, conventional dendritic cells; pDC, plasmacytoid dendritic cells; NK, natural killer cells; NKT-like, natural killer T-like cells.

To analyze the effects of MPA withdrawal on the immune cell compartments over time, PBMCs were stained with a 36-color flow cytometry panel and analyzed by spectral flow cytometry. 17 metaclusters were identified and annotated to eight major immune compartments: B cells, CD4^+^ T cells, CD8^+^ T cells, monocytes, conventional dendritic cells (cDCs), plasmacytoid dendritic cells (pDCs), natural killer (NK) cells, and natural killer T-like (NKT-like) cells (**Figures 1A, B**). The uniform manifold approximation and projection (UMAP) embedding shows the distribution of these compartments (**Figure 1A**), and their annotation was supported by expression profiles of lineage-defining markers (**Figure 1B**; **Supplementary Table 3**; **Supplementary Figures 1**, **2**).

Longitudinal mixed-effects models comparing trajectories between groups did not reveal statistically significant differences in the major immune compartments. Because this was a prospective observational cohort rather than a randomized intervention study, baseline frequencies differed between groups for some immune cell populations. To assess whether the longitudinal findings were influenced by these baseline differences, we additionally normalized each participant’s measurements to their corresponding baseline value and expressed changes at T1 and T2 as fold changes. The direction of change was consistent with the primary analyses. Detailed results are provided in **Supplementary Figure 4**. Therefore, subsequent analyses focused on longitudinal changes within individuals and the evolution of immune cell populations over time, as assessed by linear mixed-effects models, rather than on cross-sectional differences between groups at baseline or individual timepoints.

Within-group analyses demonstrated temporal changes in patients undergoing MPA withdrawal. In the MPA withdrawal group, the frequency of CD4^+^ T cells increased significantly over time (F(2, 18.9) = 3.88, *P* = 0.039), driven by a higher frequency of CD4^+^ T cells at timepoint 2 compared with baseline (estimate = 0.100, *P* = 0.020; **Figure 1C**). The frequency of CD8^+^ T cells also increased significantly over time in the MPA withdrawal group (F(2, 18.0) = 9.92, *P* = 0.001), with significant increases in frequency observed at both follow-up timepoints relative to baseline (timepoint 1: estimate = 0.033, *P* = 0.035; timepoint 2: estimate = 0.066, *P* < 0.001; **Figure 1C**).

Conversely, monocyte frequencies showed a significant decrease over time in the MPA withdrawal group (F(2, 18.1) = 5.50, *P* = 0.014), driven by a significant decrease in frequency at timepoint 2 compared with baseline (estimate = −0.060, *P* = 0.005; **Figure 1C**). B-cell frequencies showed a similar decrease over time (F(2, 17.8) = 6.25, *P* = 0.009), with significantly lower frequencies at timepoint 2 relative to baseline (estimate = −0.056, *P* = 0.003; **Figure 1C**).

In contrast, no significant temporal changes were observed within the group that continued MPA therapy (**Figure 1C**).

### 3.3 Memory T-cell subsets account for temporal changes during MPA withdrawal

**Figure 2:**
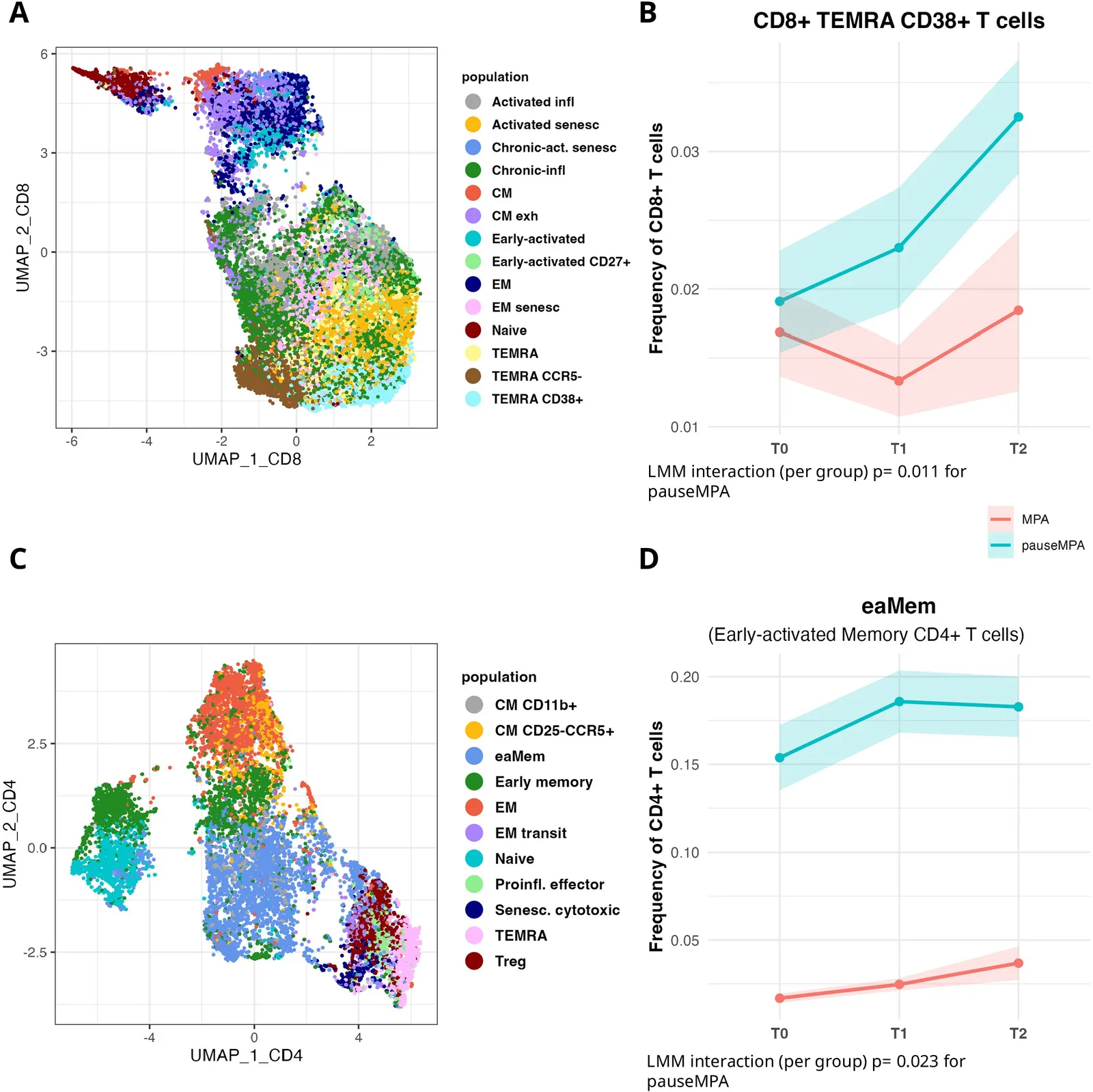
Subclustering of CD8^+^ and CD4^+^ T cells. **(A)** Uniform manifold approximation and projection (UMAP) of CD8^+^ T-cell subsets, and **(B)** longitudinal trajectories of CD8^+^ TEMRA CD38^+^ T cells in MPA continuation (red) and withdrawal (blue) groups. **(C)** UMAP visualization of CD4^+^ T-cell subsets, and **(D)** longitudinal trajectories of early-activated memory CD4^+^ T cells (eaMem) in MPA-continuation patients (red) and MPA-withdrawal patients (blue). Trajectories show mean and SEM at each timepoint per group. P values displayed in the figure indicate the overall effect of time derived from longitudinal mixed-effects models within the MPA withdrawal group. *Post hoc* pairwise comparisons to baseline are reported in the Results section. Activated infl, activated inflammatory CD8^+^ T cells; Activated senesc, Activated senescent CD8^+^ T cells; CM, central memory T cells; exh, exhausted; EM, early memory T cells; TEMRA, T cells re-expressing CD45RA; eaMem, early-activated memory CD4^+^ T cells; transit, transitory; Proinfl., proinflammatory; Treg, regulatory T cells.

To further characterize longitudinal changes in T-cell populations, CD8^+^ and CD4^+^ T cells were subclustered separately. Subsets were annotated based on their combined marker-expression profiles (**Supplementary Table 3**; **Supplementary Figure 5**) and assigned descriptive phenotypic labels corresponding to the closest previously reported T-cell populations. Subclustering of CD8^+^ T cells identified 14 distinct subsets (**Figure 2A**; **Supplementary Figure 5A**), including a CD8^+^ TEMRA subset expressing CD38. This subset demonstrated a notable effect of timepoint in the MPA withdrawal group based on longitudinal mixed-effects modeling (F(2, 17.98) = 5.79, P = 0.011), indicating temporal variation across study timepoints. *Post hoc* pairwise comparisons revealed that this effect was driven by a significant increase in frequency at timepoint 2 compared with baseline (estimate = 0.011, P = 0.005). No significant change was found at timepoint 1 (estimate = 0.002, *P* = 0.50; **Figure 2B**).

Subclustering of CD4^+^ T cells identified 11 subsets (**Figure 2C**; **Supplementary Figure 1B**), including early-activated CD4^+^ memory T cells. This subset also showed significant temporal variation within the MPA withdrawal group (F(2, 17.87) = 4.66, *P* = 0.023). *Post hoc* analyses showed higher frequencies at both timepoint 1 (estimate = 0.025, *P* = 0.033) and timepoint 2 (estimate = 0.033, *P* = 0.009) compared with baseline (**Figure 2D**).

Subclustering of B cells did not identify a distinct subset that accounted for the overall decrease in B-cell frequencies, suggesting a global effect.

### 3.4 Temporal changes in monocyte subsets during MPA withdrawal

**Figure 3:**
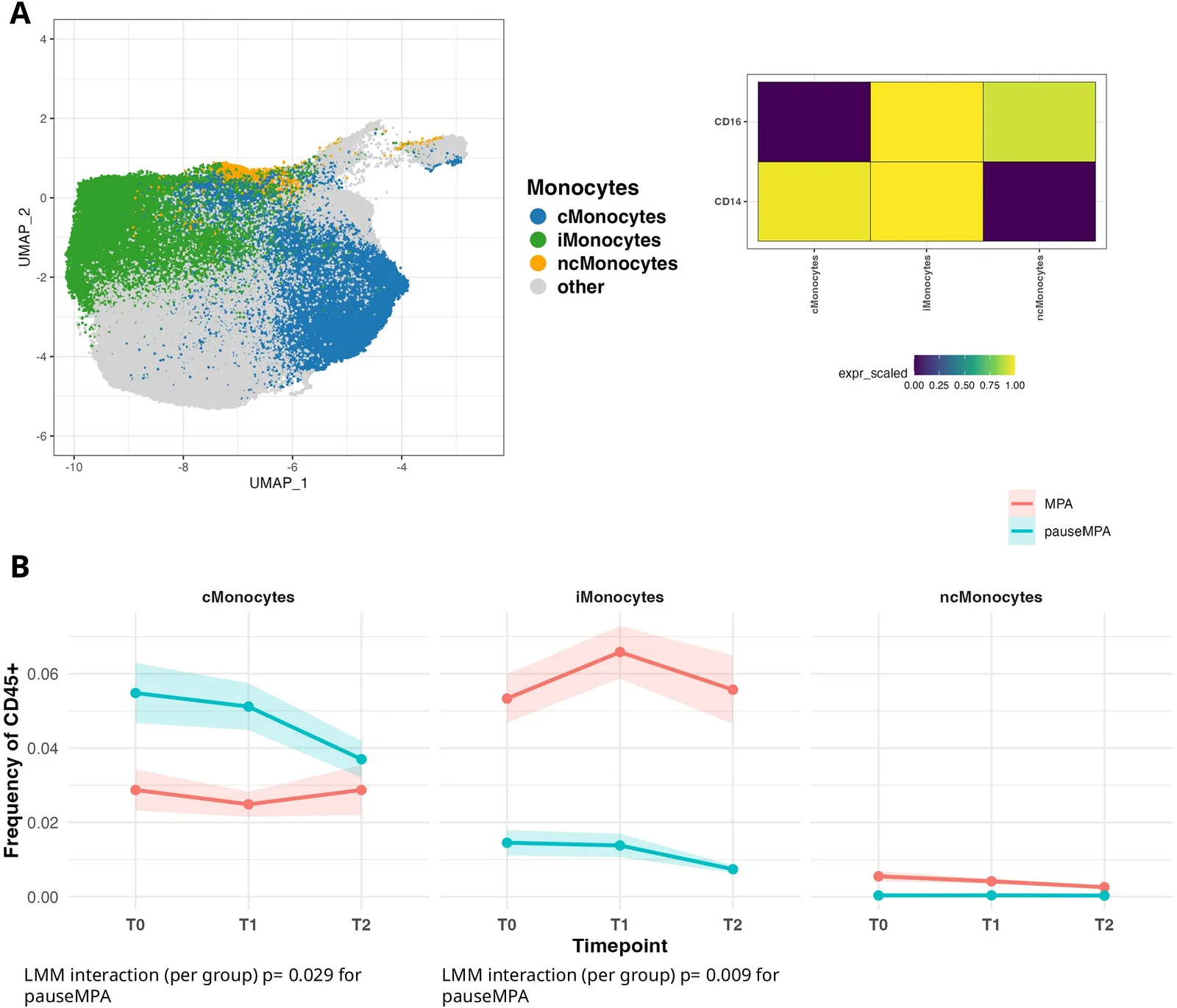
Analysis of monocyte subsets. **(A)** Color-coded Uniform manifold approximation and projection (UMAP) and heatmap showing CD14- and CD16-expression for three identified monocyte subsets. **(B)** Longitudinal trajectories (mean and SEM for each group at each timepoint) of monocyte subsets in continuation (red) and withdrawal (blue) groups. P values displayed in the figure indicate the overall effect of time derived from longitudinal mixed-effects models within the MPA withdrawal group. *Post hoc* pairwise comparisons to baseline are reported in the Results section. cMonocytes, classical monocytes; iMonocytes, intermediate monocytes; ncMonocytes, non-classical Monocytes.

Within the monocyte compartment, three subsets were identified: classical, intermediate, and non-classical monocytes (**Figure 3A**). Classical and intermediate monocytes showed significant overall effects of time in the MPA withdrawal group based on longitudinal mixed-effects models. For classical monocytes, a significant effect of time was observed (F(2, 17.75) = 4.34, *P* = 0.029). *Post hoc* pairwise comparisons revealed that this effect was driven by a significant decrease in frequency at timepoint 2 relative to baseline (estimate = −0.015, *P* = 0.010; **Figure 3B**). Similarly, intermediate monocytes showed a significant time effect (F(2, 17.83) = 6.30, *P* = 0.009). *Post hoc* analyses demonstrated significantly lower frequencies at timepoint 2 relative to baseline (estimate = −0.005, *P* = 0.004; **Figure 3B**).

No significant temporal changes were observed for non-classical monocytes in within-group analyses.

### 3.5 Time since transplantation and patient age modulate changes in the frequencies of CD8^+^ T cells and monocytes

**Figure 4:**
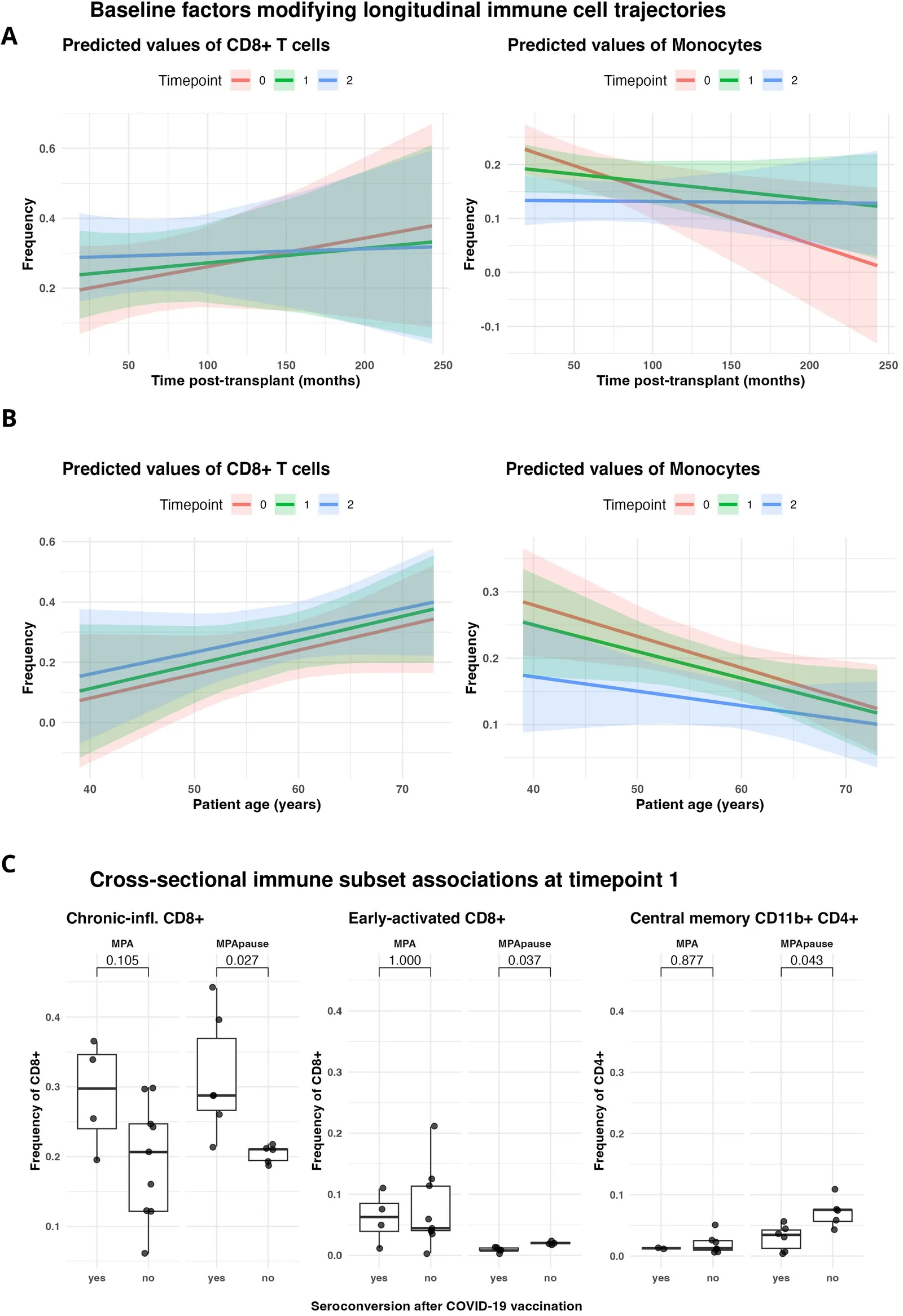
Influence of patient-specific characteristics on immune trajectories in MPA-withdrawal patients and association of cell subsets with seroconversion following COVID-19 vaccination. **(A)** Model-predicted CD8+ T cells and monocytes per timepoint across months after transplantation (TPL; shaded areas = 95% confidence intervals). **(B)** Model-predicted CD8+ T cells and monocytes per timepoint across patient age (shaded areas = 95% confidence intervals). **(C)** Boxplots showing frequencies of chronic-inflammatory (chronic-infl.) CD8^+^ T cells, early-activated CD8^+^ T cells and central memory CD11b^+^ CD4^+^ T cells at timepoint 1, separated by seroconversion status and patient group. “Yes” and “No” indicate seroconversion status following COVID-19 vaccination. P values were calculated using two-sided Wilcoxon rank-sum tests and adjusted for multiple comparisons using the Benjamini–Hochberg false discovery rate method.

In the MPA withdrawal group, interaction models incorporating timepoint and time since transplantation showed that the increase in CD8^+^ T-cell frequency was attenuated in patients with longer time since transplantation (estimate = −0.0007, interaction *P* = 0.027 at timepoint 2; **Figure 4A**). Likewise, the decrease in monocyte frequency diminished with increasing time since transplantation (interaction, *P* = 0.016; estimate = 0.0009 at timepoint 2; **Figure 4A**). Notably, patients with longer time since transplantation already exhibited significantly lower baseline monocyte frequencies (*P* = 0.013; **Figure 4A**), suggesting that these interaction effects may partly reflect pre-existing differences in immune cell composition. Older patients generally exhibited lower monocyte frequencies across all time points (age effect estimate = −0.0047 per year, *P* = 0.018), but age did not affect the direction of change after MPA withdrawal (**Figure 4B**). We did not observe a significant influence of patient age on CD8+ T-cell frequencies (**Figure 4B**), or any other cell population.

### 3.6 T-cell subset differences associated with seroconversion status after COVID-19 vaccination

To assess predictive effects on seroconversion, baseline (timepoint 0) immune cell frequencies were analyzed for their association with subsequent seroconversion. Logistic regression models revealed no significant predictive associations for any major immune compartment (all *P* > 0.29). No significant differences in major immune cell populations were observed between seroconverters and non-seroconverters. Together, these findings indicate that major circulating immune cell frequencies were not associated with seroconversion status either cross-sectionally at timepoint 1 or predictively from baseline measurements.

Within the MPA withdrawal group at timepoint 1, immune cell subsets were compared between seroconverters (*n* = 6) and non-seroconverters (*n* = 5). Among CD8^+^ T cells, chronic inflammatory subsets were present at significantly higher frequencies in seroconverters (*P* = 0.027; **Figure 4C**), while early activated subsets were less frequent (*P* = 0.037; **Figure 4C**). Within the CD4^+^ compartment, central memory CD11b^+^ CD4^+^ T cells were also present at significantly lower frequencies in seroconverters (*P* = 0.043; **Figure 4C**), whereas no significant differences were observed for other CD4^+^ subsets. No significant differences were detected across B-cell subsets.

Together, these exploratory findings suggest that seroconversion following MPA withdrawal and COVID-19 vaccination may be associated with qualitative differences in T-cell activation and differentiation rather than global alterations in major immune compartments. However, these observations should be interpreted cautiously, given the limited sample size and exploratory nature of the analyses.

## 4 Discussion

In this study, we provide a detailed characterization of immune cell dynamics during short-term MPA withdrawal in KTR. Using high-dimensional spectral flow cytometry combined with unsupervised clustering, we identified temporal changes across T-cell, monocyte, and B-cell compartments, providing insight into the heterogeneous immune responses accompanying transient modulation of immunosuppression.

As this was a prospective observational cohort rather than a randomized intervention study, some immune cell populations differed between the MPA withdrawal and continuous MPA groups at baseline. Therefore, our primary objective was not to compare absolute immune cell frequencies between groups at individual timepoints, but to characterize the longitudinal evolution of immune cell populations within individuals following transient MPA withdrawal and to compare these trajectories with those observed in patients who continued MPA treatment. Accordingly, the conclusions are based on longitudinal analyses using linear mixed-effects models that account for repeated measurements within each patient.

Our findings indicate temporal changes in the frequencies of CD4^+^ and CD8^+^ T cells during short-term MPA withdrawal, including increased relative frequencies of specific subsets such as CD8^+^ TEMRA CD38^+^ cells and early-activated memory CD4^+^ T cells. These observations are consistent with the pharmacological mechanism of MPA, which selectively impairs lymphocyte proliferation by blocking *de novo* guanosine nucleotide synthesis (27). Removal of this proliferative constraint may contribute to the observed shifts in T-cell composition, including changes in populations poised for rapid effector function, such as TEMRA and early-activated memory populations (28–30). Notably, these temporal increases in relative frequency were more pronounced in patients with a shorter time since transplantation, suggesting that immune responsiveness to short-term MPA withdrawal may vary with time since transplantation.

Concurrently, we observed a significant temporal decrease in the frequencies of monocyte populations in the MPA withdrawal group, particularly in the classical and intermediate monocyte subsets. These findings suggest that short-term modulation of immunosuppression is accompanied by dynamic variation not only in adaptive but also in innate immune compartments, potentially through indirect homeostatic mechanisms. We propose that immunosuppression-induced changes in adaptive immunity may trigger compensatory shifts in innate immune cells, including monocytes, which normalize when immunosuppression is transiently reduced. Such effects have been previously described as coordinated adjustments between adaptive and innate immune compartments in transplantation settings (30, 31), including shifts toward pro-inflammatory monocyte phenotypes following transplantation and sustained immunosuppression (30). These observations support the concept of an integrated immune network in which innate and adaptive arms are closely interconnected and may exhibit compensatory dynamics (31).

The attenuation of the decrease in monocyte frequency in patients with longer time since transplantation or older age may reflect pre-existing alterations in myeloid homeostasis due to chronic immunosuppression or age-related immune remodeling, as previously described in the context of kidney and lung transplantation (31, 32). In detail, the nonspecific pro-inflammatory response in peripheral blood monocytes has been shown to decline over time in older kidney transplant patients >50 years of age (31), in healthy individuals, and in the airways of lung transplant recipients (32).

Additionally, we observed temporal decreases in overall B-cell frequencies during MPA withdrawal without evidence of a specific subset driving this pattern. However, these findings indicate only a quantitative change in circulating B-cell frequencies during transient MPA withdrawal, without allowing conclusions about B-cell recovery or underlying mechanisms. Their lack of association with seroconversion following COVID-19 vaccination suggests that quantitative B-cell dynamics alone may not fully explain humoral responsiveness, although the small sample size and absence of functional B-cell assays preclude firm conclusions.

Interestingly, immune cell dynamics were observed as overall trends, with changes emerging more pronounced between baseline and timepoint 2 - after MPA had already been reintroduced following the short-term 5-week withdrawal. Both *in vitro* and patient data suggest that cell changes became apparent within days of MPA withdrawal, consistent with its fast-acting mechanism (17, 33, 34). Although this data is drawn from the *ex vivo* context and may not necessarily translate to *in vivo* kinetics (34), it does establish that the pharmacodynamic effect of MPA is reversible at the cellular level when the drug is no longer present, with rapid restoration of lymphocyte function after MPA withdrawal. In particular, T cells have been reported to express fewer co-stimulatory molecules and more inhibitory receptors after long-term exposure to immunosuppression, specifically in the setting of kidney transplantation, which could impose a latency on their expansion (35–37). Prior studies also indicate that prolonged immunosuppression can prolong the time required for immune restoration, with full recovery of T-cell function and purine metabolism often taking weeks to months after discontinuation of immunosuppression in specific settings (38–40). Additionally, at timepoint 1, precursors of the cell populations that subsequently showed increased relative frequencies may already have been present, but the relevant reference markers were not included in our panel, and these populations might be difficult to analyze in the peripheral blood.

Exploratory analyses of immune subsets at timepoint 1 in the MPA withdrawal group suggested differences between seroconverters and non-seroconverters in selected T-cell populations. Seroconverters exhibited higher frequencies of chronic inflammatory CD8^+^ T-cell subsets and lower frequencies of early activated CD8^+^ and central memory CD11b^+^ CD4^+^ T cells. These findings may reflect qualitative differences in T-cell activation and differentiation states at the time of vaccination rather than global quantitative differences in major immune compartments, potentially indicating more effective effector maturation or a more rapid contraction of early activation states in vaccine responders. However, the phenotypic markers included in our panel, the available sampling timepoints, and the limited sample size do not provide sufficient temporal or functional resolution to distinguish between these mechanisms. Future studies with denser longitudinal sampling and functional characterization will be required to elucidate the underlying T-cell kinetics. Importantly, no immune cell subset demonstrated predictive value for seroconversion following COVID-19 vaccination in regression analyses, and no differences were observed in B-cell subsets. Together, these observations suggest that vaccine responsiveness in this setting may be influenced by complex immune functional states rather than by individual cell subset frequencies alone.

Interpretation of the identified T-cell subsets warrants some caution. Although FlowSOM enables unbiased identification of phenotypically distinct cell populations, their annotation depends on the marker panel included in the analysis. Consequently, subset names represent descriptive phenotypic labels corresponding to the closest previously described populations rather than definitive functional classifications. This is particularly relevant for activation states, which are increasingly recognized as existing along a continuum rather than as discrete biological entities. Future studies incorporating broader functional marker panels or multimodal approaches will further refine these phenotypic assignments.

Our study has several important implications. First, short-term MPA withdrawal was associated with temporal changes in the immune landscape, including increases in circulating T-cell frequencies and decreases in the frequencies of monocyte subsets and B cells, without evidence of short-term acute adverse events. This supports the clinical feasibility of transient immunosuppression modulation as a strategy to enhance vaccine responses, while potential effects on infection-related clinical outcomes require further investigation (17, 20, 21). Second, the results underscore the heterogeneity of immune reconstitution in KTR, influenced by both, time since transplantation and patient age, which may inform individualized approaches for immunomodulatory interventions. Finally, the use of unsupervised high-dimensional profiling allowed for robust identification of both major immune compartments and rare or atypical subsets, highlighting its utility in mechanistic transplantation studies.

An important aspect to consider, although beyond the scope of the present analysis, is the potential relevance of an increased CD8^+^ TEMRA frequency for rejection risk (41). In our cohort, no acute rejections were observed; however, previous work, including our own recent study in operationally tolerant KTR (42), identified an unfavorable balance between CD8^+^ TEMRA T cells and regulatory subsets (Tregs, Bregs) as a feature associated with poor graft outcomes. Operationally tolerant patients, by contrast, displayed low TEMRA frequencies and high frequencies of regulatory populations, reflected in low TEMRA-to-Treg/Breg ratios (42). Thus, the temporal increase in the frequency of CD8^+^ TEMRAs observed during MPA withdrawal may reflect transient shifts in the effector/regulatory balance and warrant careful monitoring in larger cohorts with longer follow-up. The CD38 expression detected within these subsets may further raise mechanistic questions, given that CD38^+^ cells have been implicated in antibody-mediated rejection, although NK cells rather than T cells were suggested as key drivers in recent studies (41, 43–45). Together, these findings argue for future work to clarify whether transient MPA withdrawal, while clinically safe in the short term, could alter the regulatory/effector equilibrium in ways that affect long-term graft stability.

Limitations of our study include the relatively small sample size, the observational design within a single-center cohort, and the short-term follow-up, which preclude conclusions regarding long-term graft outcomes, infection-related clinical outcomes, or the relative contribution of vaccination and temporary MPA withdrawal to the observed immune changes. Furthermore, because all participants received SARS-CoV-2 vaccination during the study period, the effects of temporary MPA withdrawal cannot be fully disentangled from vaccine-induced immune responses. Although the inclusion of a vaccinated control group that continued MPA therapy allowed comparison of longitudinal immune trajectories under different immunosuppressive regimens, our study was not designed to determine the antigen specificity of the immune cell populations showing increased relative frequencies. In particular, whether the observed increase in the relative frequency of CD38^+^ CD8^+^ T cells reflects SARS-CoV-2-specific responses remains unknown. Future studies combining antigen-specific approaches, such as peptide-MHC tetramer staining or T-cell receptor repertoire sequencing, with longitudinal immune profiling will be required to address this question. Evidently, larger cohorts are needed to validate these findings and explore the clinical relevance of specific immune subset changes for both vaccine responsiveness and allograft health. Importantly, our findings should not be interpreted as supporting long-term MPA withdrawal, since MPA continues to play an essential role in immunosuppressive regimens and is supported by substantial evidence for its efficacy in reducing rejection and improving graft survival (46, 47).

In summary, our results show that short-term MPA withdrawal is associated with temporal changes in immune cell composition, including increases in CD4^+^ and CD8^+^ T-cell frequencies and decreases in the frequencies of monocytes and B cells in KTR. These longitudinal patterns varied according to time since transplantation and patient age, highlighting interindividual variability in immune cell dynamics. High-dimensional immune profiling provided detailed characterization of these dynamic changes and may support future efforts to better understand immune adaptation during transient immunosuppression modulation in transplantation.

## Data Availability

The raw data supporting the conclusions of this article will be made available by the authors, without undue reservation.
