# Supplementary material for: Short-term mycophenolic acid withdrawal is associated with temporal changes in immune cell composition in mRNA-1273-vaccinated kidney transplant recipients: insights from high-dimensional immune profiling
Source: Front Immunol. 2026 Aug 17;17:1826672. doi: 10.3389/fimmu.2026.1826672 (PMC13526548; doi:10.3389/fimmu.2026.1826672)
Supplement: Supplementary file 1 [file DataSheet1.pdf]

Supplementary Material

Table of Contents

Supplementary Methods

Supplementary Table 1 Reproducibility of repeated FlowSOM clustering based on coefficients of variation of annotated immune cell populations

Supplementary Table 2 Number of cells available and included in the FlowSOM analysis for each patient sample

Supplementary Table 3 Markers used for clustering and subset annotation

Supplementary Figure 1 FlowSOM spanning tree and marker expression heatmap

Supplementary Figure 2 Annotation of FlowSOM metaclusters

Supplementary Figure 3 Total leukocyte counts and exploratory leukocyte-scaled estimates of major immune cell populations

Supplementary Figure 4 Baseline-normalized changes in major immune cell populations

Supplementary Figure 5 Annotation of T-cell subsets

Supplementary Methods

2.2 PBMC Isolation

PBMCs were isolated from peripheral blood using Ficoll density gradient centrifugation under sterile conditions at the Department of Nephrology, Heidelberg University Hospital. Whole blood was diluted 1:1 with sterile PBS (Sigma-Aldrich, St.Louis, MO, USA) and layered onto Leucosep tubes (Greiner Bio-One, Frickenhausen, Germany) prefilled with Ficoll-Paque (GE Healthcare, Chicago, IL, USA). After centrifugation at 2000 rpm for 25 minutes, the PBMC-containing interphase was carefully collected.

Cells were washed twice with PBS (Sigma-Aldrich) (1400 rpm, 10 minutes) and resuspended in RPMI 1640 medium (Sigma-Aldrich). Cell viability and concentration were determined using trypan blue (Sigma-Aldrich) exclusion in a Neubauer counting chamber.

PBMCs were cryopreserved at a concentration of $\geq 10 \times 10^6$ cells/mL in ibidi Cryo Medium (ibidi, Gräfelfing, Germany) and stored at –80°C overnight in a controlled-rate freezing container (Mr. Frosty, Thermo Fisher Scientific, Waltham, MA, USA) before transfer to liquid nitrogen for long-term storage.

Supplementary Table 1 Reproducibility of repeated FlowSOM clustering based on coefficients of variation of annotated immune cell populations

Population	Mean frequency	SD frequency	CV	CV percent
CD4	0.210	0.000991	0.00471	0.471
CD8	0.218	0.00328	0.0150	1.50
Monocytes	0.330	0.00213	0.00646	0.646
B cells	0.0114	0.000559	0.0490	4.90
CD8+ TEMRA CD38+	0.0396 *	0.00249	0.0629	6.29
CD4+ early-activated memory	0.0532 *	0.000991	0.0465	4.65

Reproducibility of repeated FlowSOM clustering analyses. The table summarizes the mean frequency, standard deviation (SD), and coefficient of variation (CV) of the manually annotated FlowSOM-derived immune cell populations across five independent clustering runs performed using identical input data and analysis parameters while varying only the random seed.

Supplementary Table 2 Number of cells available and included in the FlowSOM analysis for each patient sample

Sample ID	Total cells after preprocessing	Cells included in FlowSOM
299 T0.fcs	8487	8487
299 T1.fcs	30403	10000
322 T1.fcs	36020	10000
298 T0.fcs	42969	10000
052 T0.fcs	43397	10000
176 T0.fcs	45588	10000
298 T2.fcs	66666	10000
347 T0.fcs	69767	10000
328 T2.fcs	89295	10000
344 T0.fcs	105043	10000
215 T0.fcs	119928	10000
347 T2.fcs	131572	10000
052 T2.fcs	137427	10000
203 T0.fcs	146318	10000
354 T1.fcs	204094	10000
328 T1.fcs	211795	10000
270 T1.fcs	234296	10000
096 T1.fcs	257390	10000
270 T0.fcs	262511	10000
176 T1.fcs	297946	10000
116 T0.fcs	325429	10000
328 T0.fcs	373656	10000
322 T0.fcs	383008	10000
170 T1.fcs	425826	10000
HC B062.fcs	449314	10000
170 T0.fcs	450404	10000
052 T1.fcs	451484	10000
203 T1.fcs	471369	10000
298 T1.fcs	474759	10000
167 T1.fcs	480569	10000
171 T2.fcs	482068	10000
167 T0.fcs	501976	10000
347 T1.fcs	520325	10000
HC B087.fcs	530521	10000
344 T1.fcs	538282	10000
344 T2.fcs	546927	10000
231 T0.fcs	576107	10000
256 T0.fcs	588682	10000
171 T1.fcs	646585	10000
256 T1.fcs	647420	10000
322 T2.fcs	675904	10000
190 T0.fcs	726314	10000
096 T0.fcs	800553	10000
210 T1.fcs	826549	10000
176 T2.fcs	895600	10000
HC P010.fcs	928418	10000
HC B039.fcs	939583	10000
116 T1.fcs	972775	10000
129 T1.fcs	1000000	10000
129 T2.fcs	1000000	10000
151 T0.fcs	1000000	10000
151 T1.fcs	1000000	10000
151 T2.fcs	1000000	10000
167 T2.fcs	1000000	10000
190 T1.fcs	1000000	10000
190 T2.fcs	1000000	10000
203 T2.fcs	1000000	10000
210 T0.fcs	1000000	10000
210 T2.fcs	1000000	10000
215 T1.fcs	1000000	10000
215 T2.fcs	1000000	10000
231 T1.fcs	1000000	10000
231 T2.fcs	1000000	10000
270 T2.fcs	1000000	10000
276 T0.fcs	1000000	10000
276 T1.fcs	1000000	10000
276 T2.fcs	1000000	10000
354 T2.fcs	1000000	10000
HC P065.fcs	1000000	10000

Total number of cells available per sample and number of cells included in the FlowSOM analysis after random downsampling. Samples were downsampled to ensure equal cell representation across samples prior to unsupervised clustering.

Supplementary Table 3 Markers used for clustering and subset annotation

Fluorochrome	Marker	Major Immune Compartments	CD4+ T cells	CD8+ T cells	B cells
BUV395	CD45RA		x	x	
BUV496	CD16	x			
BUV563	CCR5		x	x	
BUV661-A	CD11c	x			x
BUV737	CD56	x			
BUV805	CD8	x			
BV421	CCR7		x	x	
Super Bright 436	CD123	x			
BV480-A	IgD				x
BV510	CD3	x			
Pacific Orange-A	CD20	x			
BV570-A	IgM				x
BV605-A	IgG				x
BV650	CD28		x	x	
BV711-A	CCR6				x
BV750-A	CXCR5				x
BV785	PD1		x	x	x
FITC	CD57		x	x	
Spark Blue 550	CD14	x			
PerCP	CD45	x			
PerCP-Cy5.5	CD11b		x	x	
PE	CD25		x	x	
cFluor YG584	CD4	x			
PE-Dazzle594-A	CD24				x
PE-Cy5	CD95		x	x	x
PE-Cy7	CXCR3		x	x	x
APC	CD27	x	x	x	x
Alexa Fluor 647	CD1c	x			
Spark NIR 685	CD19	x			
APC-R700	CD127		x	x	
APC-Fire 750	HLADR	x	x	x	x
APC-Fire 810	CD38		x	x	x

List of markers included in the respective clustering analyses (major immune compartments, $CD4^+$ T cells, $CD8^+$ T cells, and B cells) and their use for subset identification.

Supplementary Figure 1 FlowSOM spanning tree and marker expression heatmap

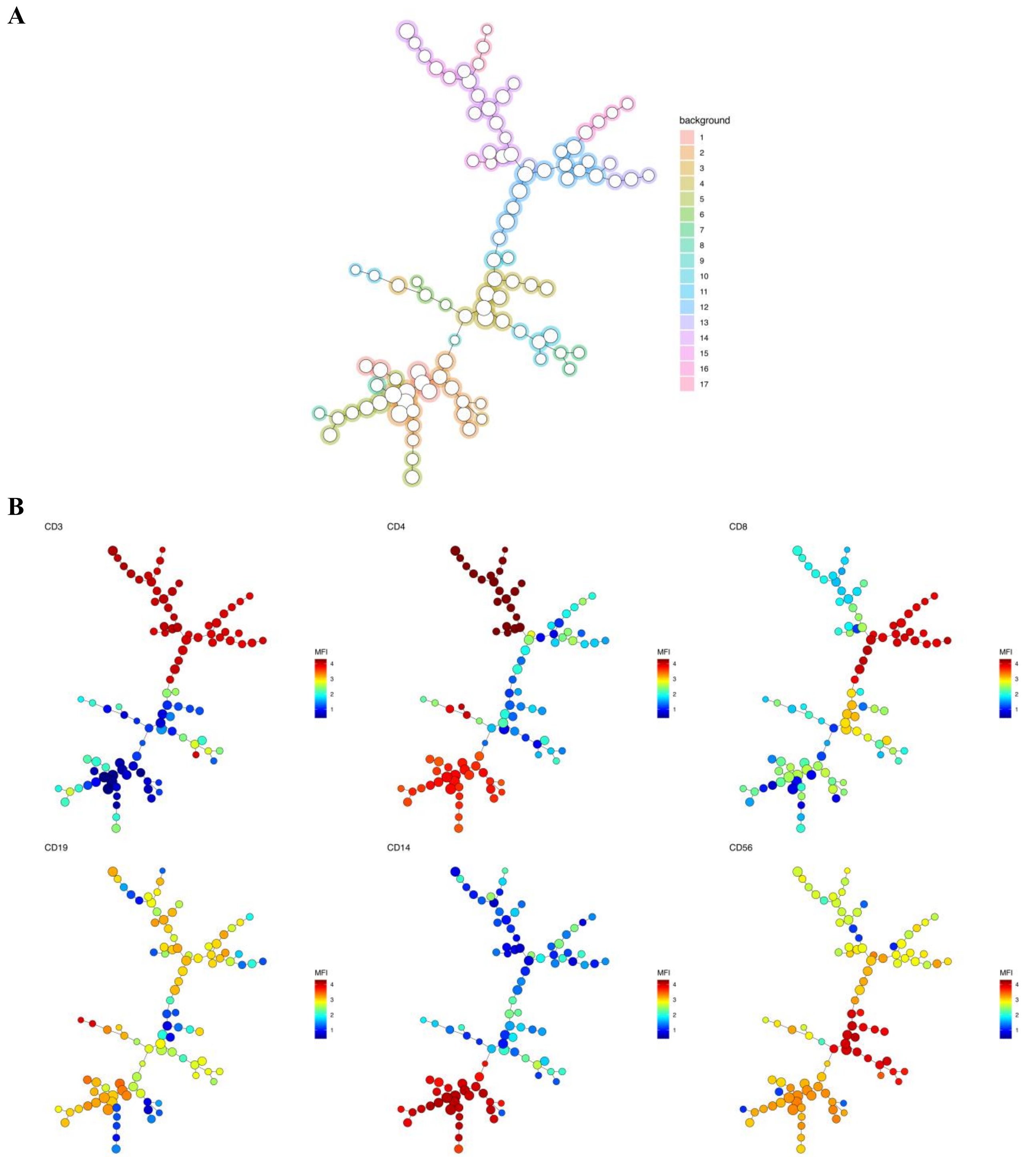

(A) FlowSOM spanning tree generated from spectral flow cytometry data showing the organization of all self-organizing map nodes. Nodes are colored according to the assigned metaclusters (1–17). (B) Median fluorescence intensity (MFI) of representative lineage markers projected onto the FlowSOM spanning tree. Marker expression of CD3, CD4, CD8, CD19, CD14, and CD56 illustrates the phenotypic distribution of T cells, B cells, monocytes, and NK cells across the identified metaclusters and served as the basis for subsequent cluster annotation.

Supplementary Figure 2 Annotation of FlowSOM metaclusters

A

B

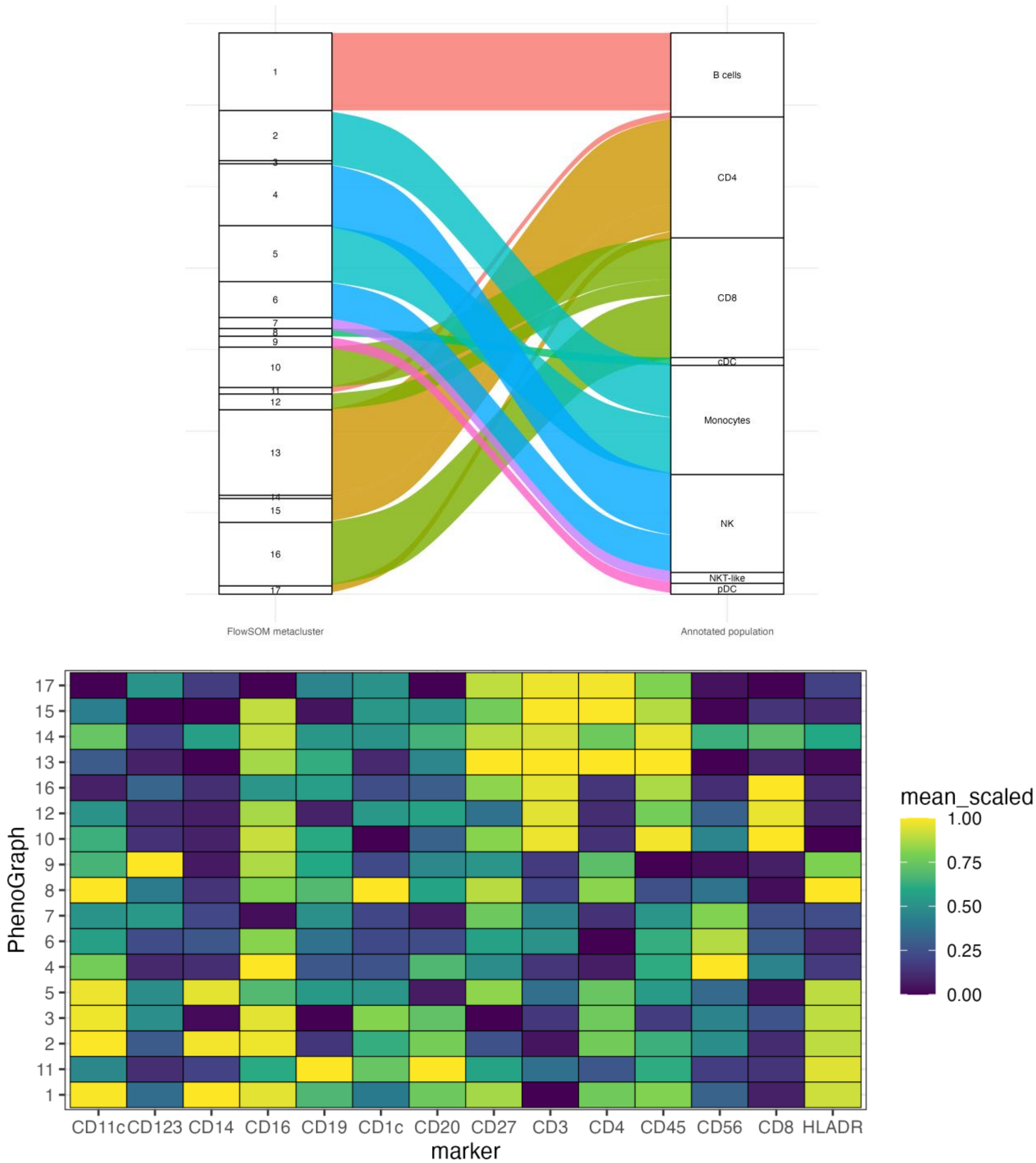

(A) Alluvial plot illustrating the correspondence between the 17 metaclusters and the manually annotated major immune cell populations. The width of each connection is proportional to the number of cells assigned from each metacluster to the respective immune cell population.
(B) Heatmap displaying scaled median expression of all markers included in the clustering panel across the 17 FlowSOM metaclusters. Marker expression profiles were used to assign metaclusters to biologically defined immune cell populations, including B cells, CD4^{+} T cells, CD8^{+} T cells, conventional dendritic cells (cDCs), monocytes, NK cells, NKT-like cells, and plasmacytoid dendritic cells (pDCs).

Supplementary Figure 3 Total leukocyte counts and exploratory leukocyte-scaled estimates of major immune cell populations

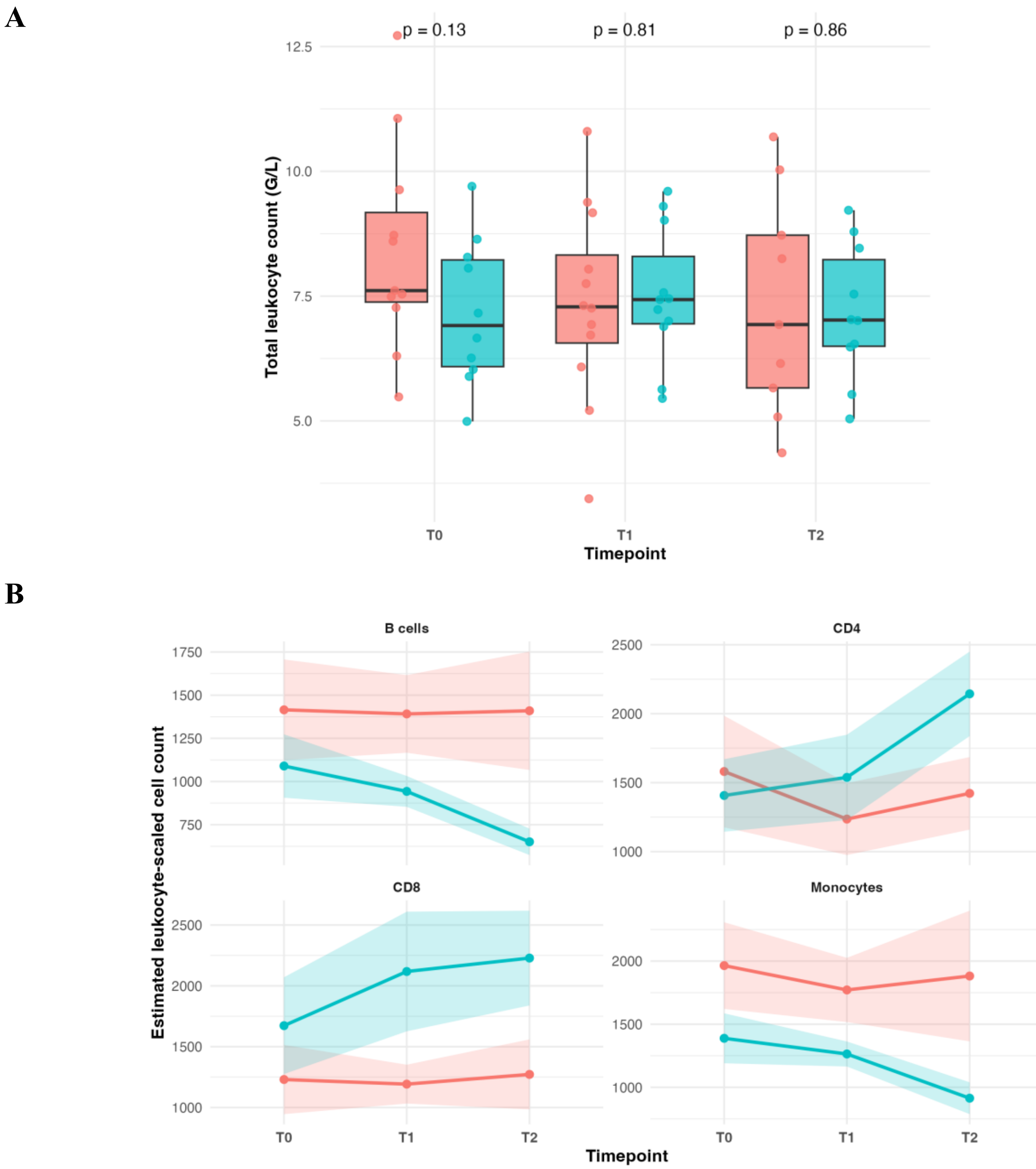

(A) Total peripheral leukocyte counts at baseline (T0), one month (T1), and three months (T2) in patients continuing MPA and patients undergoing temporary MPA withdrawal. Points represent individual patients, and boxplots show the median and interquartile range. P values indicate comparisons between the immunosuppression groups at each timepoint.

(B) Exploratory leukocyte-scaled estimates of B cells, $CD4^+$ T cells, $CD8^+$ T cells, and monocytes over time in both immunosuppression groups. Estimates were calculated by multiplying the relative frequency of each PBMC population by the corresponding total peripheral leukocyte count. Points and lines represent group means, and shaded areas represent the standard error of the mean (SEM). Because cytometry was performed on isolated PBMCs, whereas total leukocyte counts also include granulocytes, these estimates should not be interpreted as validated absolute cell counts.

Supplementary Figure 4 Baseline-normalized changes in major immune cell populations

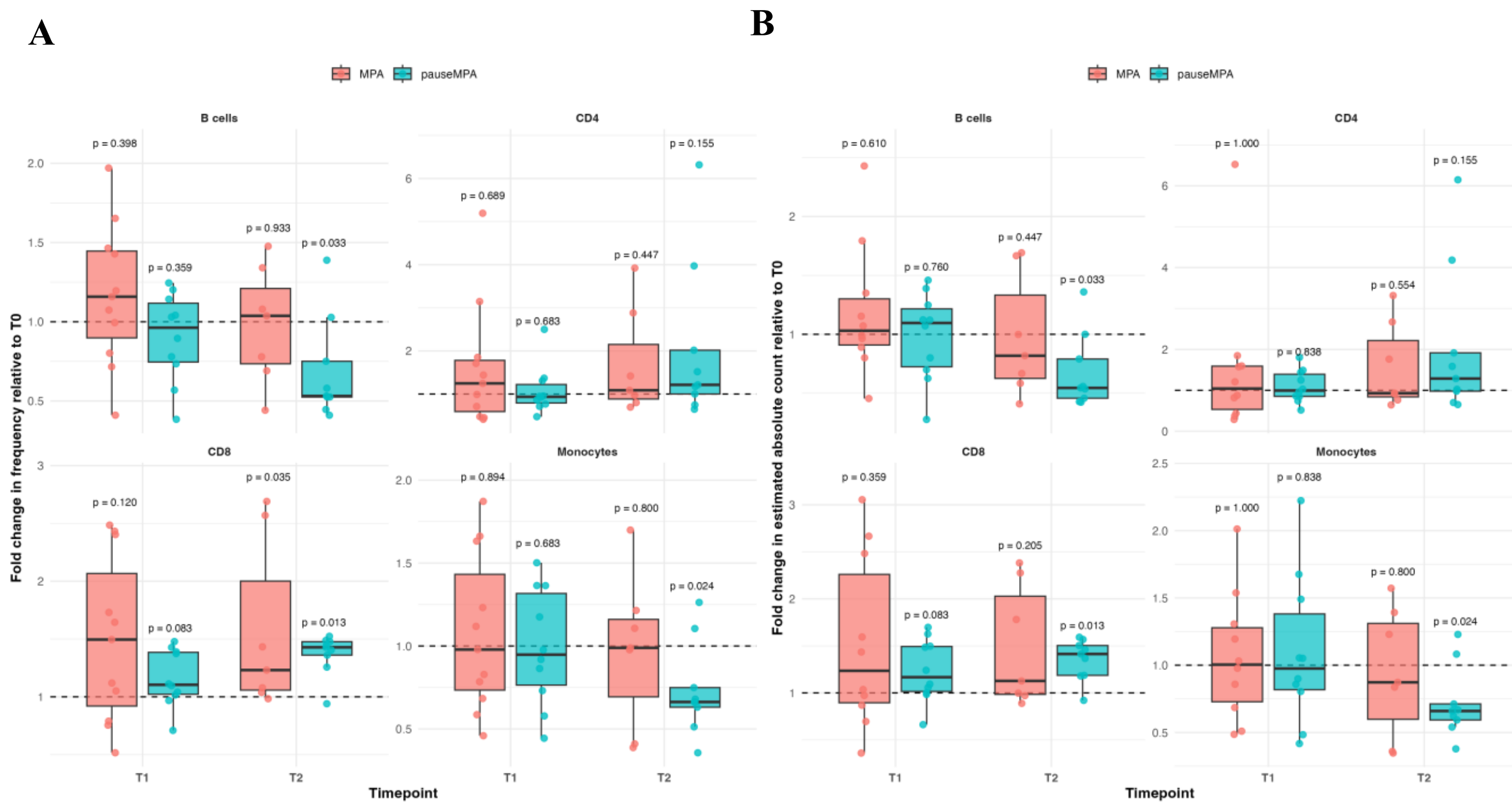

(A) Fold changes in the relative frequencies of B cells, $CD4^{+}$ T cells, $CD8^{+}$ T cells, and monocytes at one month (T1) and three months (T2), normalized to each patient's corresponding baseline value (T0), in patients continuing MPA and patients undergoing temporary MPA withdrawal. The dashed horizontal line indicates no change from baseline (fold change = 1). Points represent individual patients, and boxplots show the median and interquartile range. P values indicate within-group comparisons with baseline.

(B) Fold changes in exploratory leukocyte-scaled estimates of B cells, $CD4^{+}$ T cells, $CD8^{+}$ T cells, and monocytes at T1 and T2, normalized to each patient's corresponding baseline value. Estimated cell counts were calculated by multiplying the relative frequency of each PBMC population by the corresponding total peripheral leukocyte count. The dashed horizontal line indicates no change from baseline (fold change = 1). Points represent individual patients, and boxplots show the median and interquartile range. P values indicate within-group comparisons with baseline. Because cytometry was performed on isolated PBMCs, whereas total leukocyte counts also include granulocytes, these estimates should not be interpreted as validated absolute cell counts.

Supplementary Figure 5 Annotation of T-cell subsets

A Heatmap annotation CD8+ Tcells

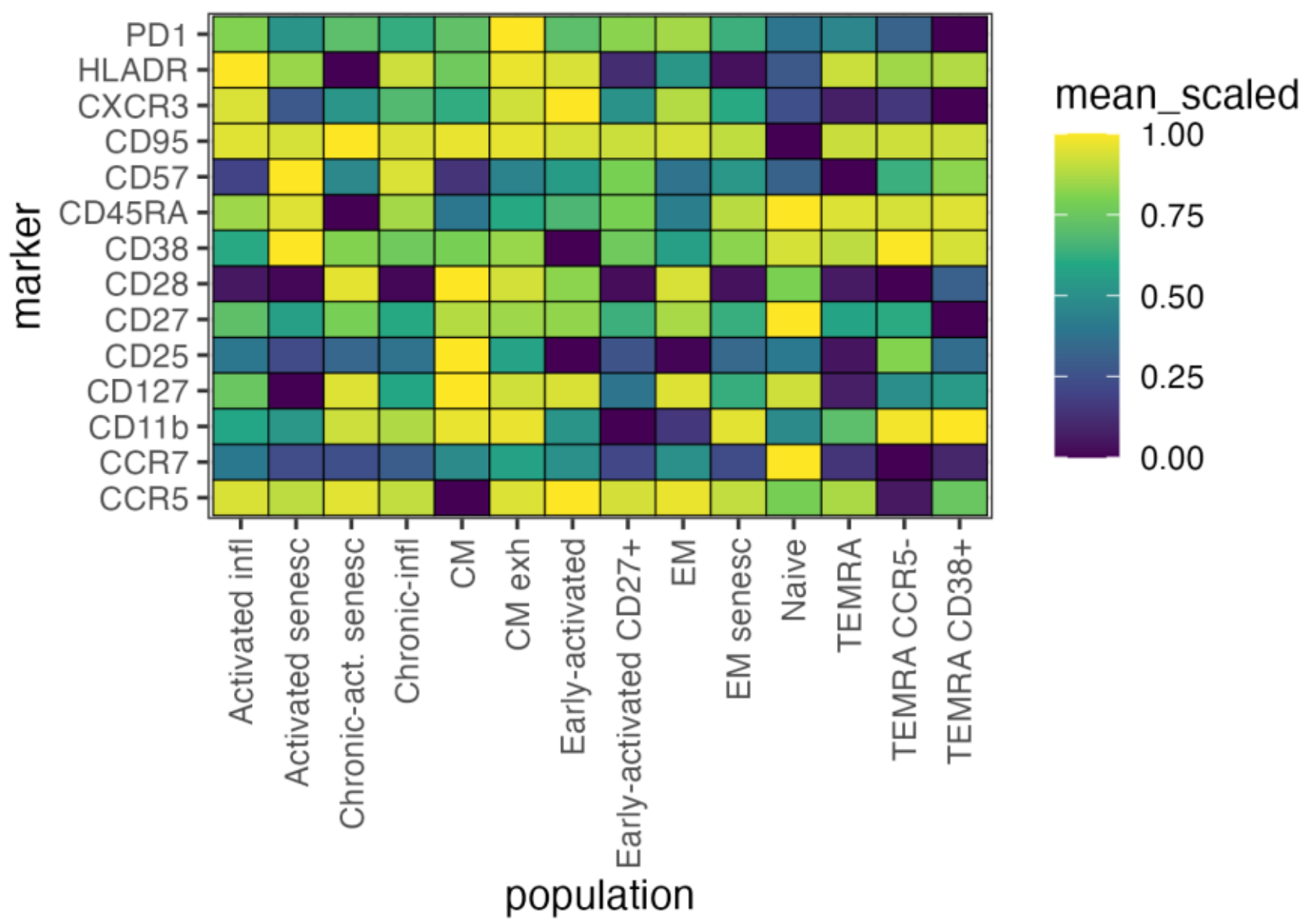

B Heatmap annotation CD4+ Tcells

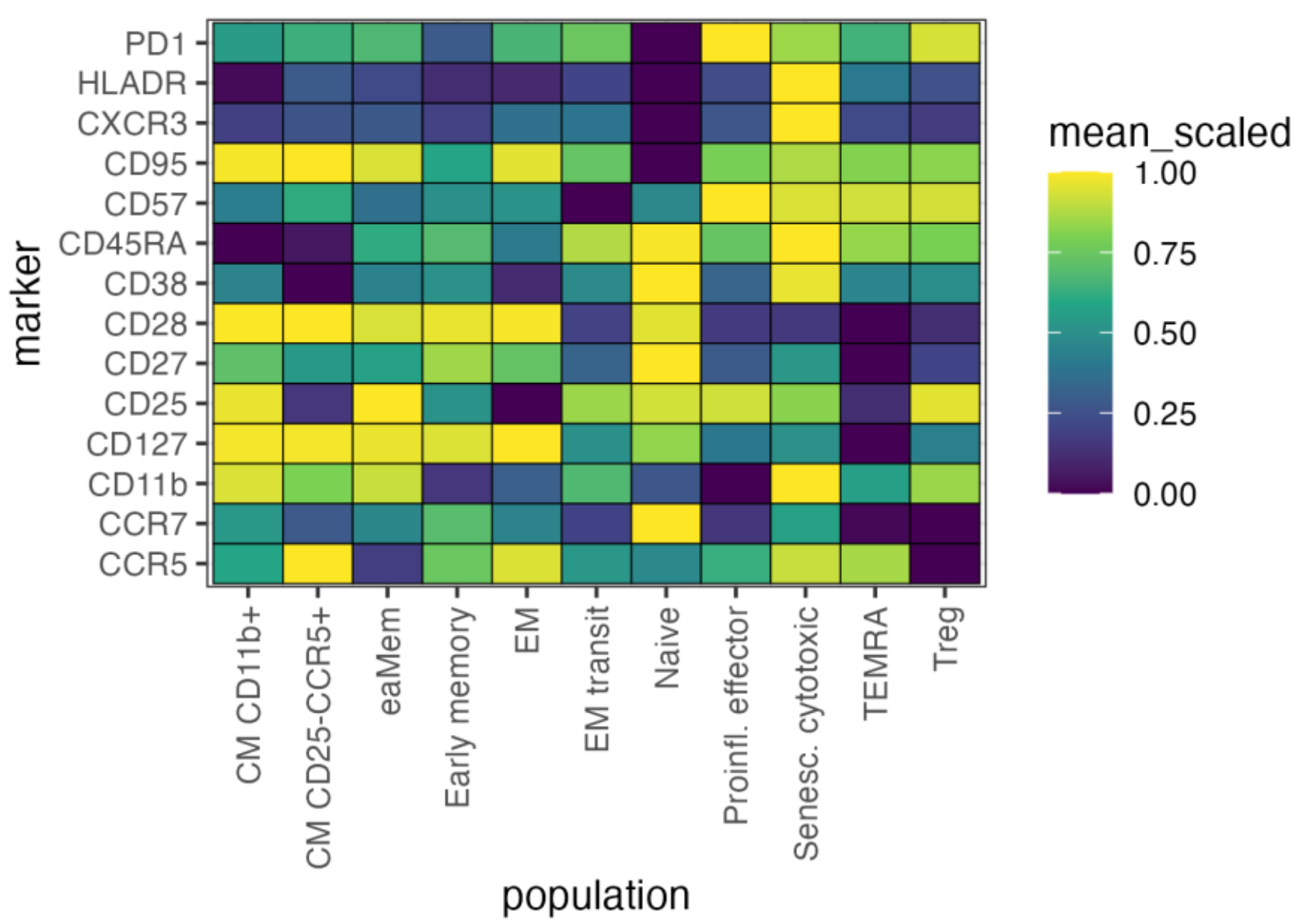

(A) Heatmap showing scaled marker expression used for the annotation of CD8^{+} T-cell subsets. The x-axis indicates subset names, and the y-axis lists the corresponding markers. Colors represent scaled expression levels. (B) Heatmap showing scaled marker expression for the annotation of CD4^{+} T-cell subsets, displayed as in panel A.
